# Letter from Oakland, Md.

**Published:** 1877-09

**Authors:** 


### EDITORIAL, ETC.

---

*Letter from Oakland, Md.*—

OAKLAND, MD., AUGUST 14, 1877.

*My Dear Dr.*—I left you on yesterday, with a promise that I would make a note of whatever was of interest at the Joint Dental Convention, convening at this place to-day, and I now try to redeem that promise so far as I am able, premising that, as I have been up o'nights, and have enough malarial fever to make all my bones ache and my head to swim, you will not expect a great deal.

Your readers will all understand that three distinct Conventions or Associations were appointed to meet here: The American Dental Convention, Southern Dental Association, and the Maryland and District of Columbia Dental Society, and that the gathering partakes more of the nature (to use the language of Dr. Atkinson,) of a "mass meeting" than of a more regular organization.

The American Dental Convention was the original organization that gave birth to the American Dental Association, and which has still existed, its offspring meantime outstripping it in growth and in the interest which clusters around it, and whose members as a whole, deem the parent body, if not obsolete, at least of undesirable existence. The American Dental Convention on the other hand, holding its original organization intact and separate ; and a spirit of something akin to jealousy is plainly apparent in the attitudes of both, the original body languishing somewhat, the junior organization strong and vigorous.

The Southern Dental Association, your readers are familiar with. Organized at a time when such a union of the Dentists of the South seemed desirable, when sectional feeling north of the Potomac made it uncomfortable for Southern men to witness the exaggerations of loyalty which were naturally manifested by the victors in so fierce a struggle, the first few meetings of this body were equal in point of strength and talent to any gatherings of the sort in the land. For several years the interest seemed unabated, but lately, the wide area over which the membership were scattered, the difficulties and cost of travel, and above all the growing poverty of our Southern States have caused a falling off in the membership, until for the past two or three years no quorum has been in attendance. The Annual Meeting that was to have convened in Montgomery, Ala., in May last, was adjourned at the instance of the executive committee, to meet at this place and time in conjunction with the above named associations.

The Maryland and District of Columbia Society, is a comparatively new organization, and includes among its members a number of the Dentists of Baltimore and Washington, and seems complete and compact in its organization and of very fair working quality.

The American Dental Convention is presided over by Dr. W. H. Atkinson, of N. Y., so well known as to need no introduction ; the Southern Association by Dr. Arthur C. Ford, of Atlanta, Ga., Vice President of that Association, and the Maryland and District of Columbia Society, by Dr. R. Finley Hunt, of Washington, D. C.

The three bodies meet together in one building, and in joint session, are permitted to debate most questions which arise in any of the societies, and most are members of each and all the bodies.

Dr. Atkinson is usually the presiding officer, and I may add, the master spirit of the meeting ; "the lungs of the affair," so one member said, but this was hardly fair of the good doctor, who whatever else may be said of him, can state a question with as much clearness and perspicuity as any one, and who presides with a great deal of fairness, justness and good temper. If the organization of all of our brains is not such as to follow the Dr. through his convoluted sentences to their terminations it is perhaps our misfortune.

I am pained to see no greater number in attendance than I do here. Of the far Southern men, I see only Ford and Whittaker, of Ga., and Walker, of La; from Virginia, Steele, and Mahoney of Richmond; while some eight or ten from Washington, D. C., and as many from Baltimore, with a sprinkling from Maryland, outside of Baltimore, and a few from Pittsburg and also Prof. J. Taft, of Cincinnati, make up the attendance—in all about thirty. This is the first day however, and to-morrow it is expected that a larger number will appear.

We are here 2700 feet above tide water, in the heart of the Alleghanies—a delightful resort.

The business of the Joint Meeting was inaugurated by a brief address from Dr. Atkinson, setting forth the original aims of the American Dental Convention, the attempts at merging it into other Associations, the difficulties attending the efforts at establishing one great National Association, and the present status of the convention and its attitude toward its rival, the American Dental Association. He himself had accepted the position of President in the belief that something might be done in that capacity by him toward healing the breach, and making at least friendly terms between these antagonistic elements.

No other business was done on the morning of the first day, save the organizing of the bodies and this address, and at its conclusion the meeting adjourned until the afternoon

I notice among those in attendance, several gentlemen not dentists, who promise to appear conspicuously in the doings of this meeting,—Dr. S. Waterman, of N. Y., well known as a spectroscopist, and Dr. Caldwell, of Baltimore, who has made electricity a specialty. Dr. Waterman has promised a paper, with illustrations, on the Spectroscope, which will be worth hearing, as it has special bearing on the subject of anæsthesia, and the changes wrought in the blood by these agents.

But as this preliminary letter is already long, I will stop.

